# Prevalence of A2143G mutation of *H. pylori-23S rRNA *in Chinese subjects with and without clarithromycin use history

**DOI:** 10.1186/1471-2180-8-81

**Published:** 2008-05-28

**Authors:** Zhuoqi Liu, Jing Shen, Lian Zhang, Lin Shen, Qiang Li, Baozhen Zhang, Jing Zhou, Liankun Gu, Guoshuang Feng, Junling Ma, Wei-Cheng You, Dajun Deng

**Affiliations:** 1 Peking University School of Oncology and Beijing Institute for Cancer Research and Beijing Cancer Hospital, Hai-Dian District, Beijing, 100036, PR China; 2 Nanchang University Medical College, Nanchang, 330006, PR China

## Abstract

**Background:**

A2143G mutation of *23S rRNA *gene of *H. pylori *results in clarithromycin (CLR) resistance. To investigate the prevalence of the CLR resistance-related A2143G mutation of the *H. pylori*-specific *23S rRNA *gene in Chinese subjects with and without CLR use history, 307 subjects received the treatment with amoxicillin and omeprazole (OA) and 310 subjects received a placebo in 1995, and 153 subjects received a triple therapy with OA and CLR (OAC) in 2000. DNA was extracted from fasting gastric juice at the end of the intervention trial in 2003. *H. pylori *infection was determined by *H. pylori*-specific *23S rRNA *PCR, ELISA, and^13^C-urea breath test assays. Mutations of the *23S rRNA *gene were detected by RFLP assays.

**Results:**

The presence of *23S rRNA *due to *H. pylori *infection in the OA group remained lower than that in the placebo group 7.3 yrs after OA-therapy [51.1% (157/307) vs. 83.9% (260/310), p = 0.0000]. In the OAC group, the *23S rRNA *detection rate was 26.8% (41/153) three yrs after OAC-treatment. The A2143G mutation rate among the *23S rRNA*-positive subjects in the OAC group [31.7% (13/41)] was significantly higher than that in the OA group [10.2% (16/157)] and the placebo group [13.8% (36/260)]. The frequency of the AAGGG → CTTCA (2222–2226) and AACC → GAAG (2081–2084) sequence alterations in the OAC group was also significantly higher than those in the OA group and the placebo group.

**Conclusion:**

Primary prevalence of the A2143G mutation was 10~14% among Chinese population without history of CLR therapy. Administration of CLR to eliminate *H. pylori *infection increased the prevalence of the A2143G mutation in Chinese subjects (32%) significantly.

## Background

*Helicobacter pylori (H. pylori) *infection occurs worldwide. The prevalence of infection by *H. pylori *varies greatly among countries, with over a 70% infection rate in many developing countries compared to 20% to 50% in most industrialized nations. *H. pylori *infection can lead to gastritis, duodenal and gastric ulcer, gastric carcinoma, and gastric lymphoma [1-3]. Gastric cancer risks can be reduced by up to 60% by eradication of *H. pylori *infection through primary prevention, thus affecting approximately 800,000 gastric cancer patients worldwide [4,5].

Currently, a seven-day, triple-drug regimen has been recommended as one of the first-line therapies for *H. pylori *management [6,7]. This treatment includes omeprazole (a proton-pump inhibitor), clarithromycin (CLR), and amoxicillin. However, this therapy is being investigated because of increased eradication failures due to the prevalence of CLR-resistant (CLR^r^) *H. pylori *infections. Many studies have shown that between 0–50% of *H. pylori *isolates were CLR^r^, which leads to a need for long term assessment of the efficacy of CLR in the triple-drug regimen [8,9]. It is well known that the abuse of macrolide antibiotics including CLR might lead to CLR resistant forms of *H. pylori*. Whether an optimal therapy containing CLR for *H. pylori *eradication would contribute to the prevalence of CLR^r ^*H. pylori *infection was not well investigated among the Chinese population.

From 1995 to 2003, a randomized, double blind and placebo-controlled intervention trial was conducted in a high-risk population for gastric cancer in Linqu County, Shandong Province of China [10]. One of the three treatments of *H. pylori *infection in this study was a dual-drug therapy with omeprazole and amoxicillin (OA), since the triple-drug regimen was not available in 1995. This study focused on subjects who were *H. pylori *seropositive at baseline. In 2000, a triple therapy consisting of OA and CLR (OAC) was applied to subjects who were *H. pylori *seronegative at baseline and subsequently found to be ^13^C-urea breath test (^13^C-UBT)-positive, according to instruction of the Ethic Committee of the Beijing Institute for Cancer Research and the U.S. National Cancer Institute (NCI).

In *H. pylori *infection, resistance to CLR is mostly due to the presence of A2143G and A2142G/C point mutations of the *23S rRNA *gene [8,11,12]. We recently reported that the A2143G mutation was a late event in the development of CLR resistance of *H. pylori 26695 *[13]. The global average mutation frequency for the A2143G and A2142G mutations causing CLR resistance of *H. pylori *were 69.8% and 11.7%, respectively [8]. Herein, we report the prevalence of the A2143G and A2142G mutations of the *H. pylori*-specific *23S rRNA *gene among these subjects treated with regimens with or without CLR after eradication of *H. pylori *infection.

## Results

Genomic DNA was extracted from 770 of pre-fixed fasting gastric juice samples as described in the Method section. To determine both the presence of *H. pylori *infection and prevalence of CLR^r^, a *H. pylori*-specific *23S rRNA *PCR assay was developed. *H. pylori *infection was detected in 59.5% of analyzed subjects by the PCR assay as compared to 68.1% and 71.0% by ^13^C-UBT and ELISA, respectively (Table 1). If the combined results of ^13^C-UBT and ELISA assays were used as the gold standard [both positive (*H. pylori *infection) or both negative (not infected)], the PCR assay's sensitivity and specificity was 83.4% and 75.5%, respectively. Its Youdens' index was 0.59 (Table 2).

**Table 1:** Comparisons of sex, age, *H. pylori *infection, and mutations of the *23S rRNA *gene between subjects with and without CLR use history

Groups		Placebo	OA	OAC	(Total)
CLR use history		-	-	+	
Case No.		310	307	153	770
Sex ratio (M:F)		1:1.00	1:1.13	1:0.82	1:1.01
Age (yrs) (Mean ± SD)		45.3 ± 8.4	45.0 ± 8.4	45.7 ± 8.6	45.2 ± 8.5
*H. pylori*-positive rate in 2003 (%)	^13^C-UBT	90.6 (280/309)	54.1^1 ^(166/307)	53.3 (81/152)	68.1 (527/768)
	ELISA	92.5 (283/306)	62.1^1 ^(185/298)	44.1^2 ^(64/145)	71.0 (532/749)
	*23S rRNA*	83.9 (260/310)	51.1^1 ^(157/307)	26.8^2 ^(41/153)	59.5 (458/770)
Proportion of 2143G-positive *H. pylori *(%)	Total CLR^r^	13.8 (36/260)	10.2 (16/157)	31.7^3 ^(13/41)	14.2 (65/458)
	CLR^r & s^	9.6 (25/260)	6.4 (10/157)	14.6 (6/41)	9.0 (41/458)
Proportion of *MboII*-RFLP-positive *H. pylori *(%)	2222CTTCA	0.4 (1/260)	1.9 (3/157)	4.9^4 ^(2/41)	1.3 (6/458)
	2081GAAG	1.9 (5/260)	1.3 (2/157)	14.6^5 ^(4/41)	2.4 (11/458)

^1^: OA group vs. placebo group, p < 0.0000;

^2^: OAC group vs. OA group, p < 0.0005;

^3^: OAC group vs. OA group, OR = 4.09, 95%CI [1.64–10.23]; OAC group vs. placebo group, OR = 2.89, 95%CI [1.28–6.46];

^4^: OAC group vs. OA group, OR = 2.63, 95%CI [0.30–20.29]; OAC group vs. placebo group, OR = 13.28, 95%CI [0.91–379];

^5^: OAC group vs. OA group, OR = 8.38, 95%CI [1.25–68.84]; OAC group vs. placebo group, OR = 5.51, 95%CI [1.18–25.12]

**Table 2:** Comparison of accuracy of various assays for detection of *H. pylori *infection

Assays	Sensitivity	Specificity	Youden's index^1^
*23S rRNA *PCR^2^	83.4% (373/447)	75.5% (114/151)	0.59
^13^C-UBT^3^	94.9% (373/393)	70.8% (114/161)	0.66
ELISA^4^	95.2% (373/392)	63.7% (114/179)	0.59

^1^: defined as "sensitivity + specificity - 1";

^2^: both ^13^C-UBT and ELISA were positive or negative for comparison;

^3^: both PCR and ELISA were positive or negative for comparison;

^4^: both PCR and ^13^C-UBT were positive or negative for comparison.

Seven years after the OA treatment, the presence of *23S rRNA *due to *H. pylori *infection remained remarkably lower than that in the placebo group [51.1% (157/307) vs. 83.9% (260/310), p = 0.0000] (Table 1). Three years after the triple therapy with CLR, the cumulative *H. pylori *infection rate in the OAC group was 26.8% (41/153) by PCR (Table 1).

**Figure 1:**
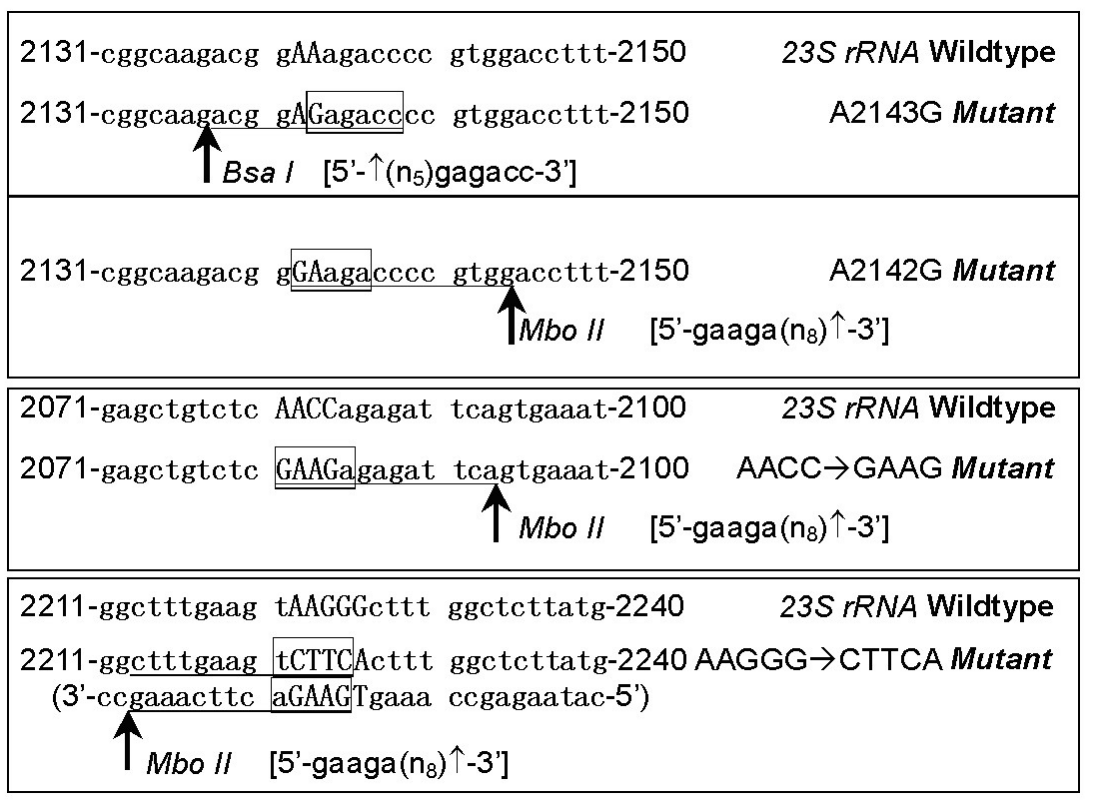
**Schematic diagram for detection of nucleotide alterations of *23S rRNA *by PCR-RFLP assays**. The *23S rRNA *sequence of *H. pylori 26695 *was illustrated as the wild-type sequence [GenBank No.: U27270].

**Figure 2:**
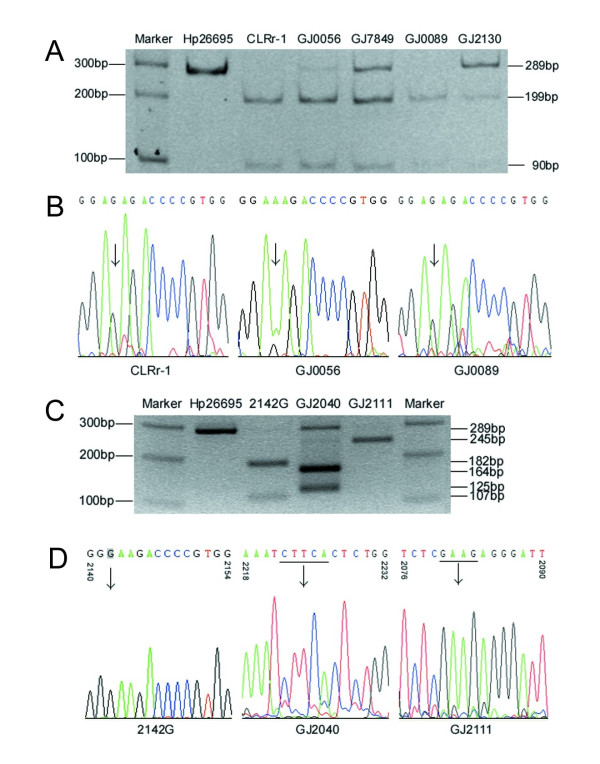
**Chromatograms of PCR-RFLP assays and sequencing for detection of nucleotide alterations of *23S rRNA***. *H. Pylori 26695 *and CLR^r^-1 were used as negative and positive control of A2143G mutation. *BsaI *digestion of the PCR products of representative samples was displayed on 8% PAGE gel. The 289 bp A2143G-positive PCR products were cleaved into a 199 bp and a 90 bp fragments (**A**). The A2143G mutation was also confirmed by sequencing of the PCR products of *23S rRNA *(**B**, displayed 2140–2154 fragment). *H. Pylori 26695 *and a *2142G *clone were used as negative and positive control of A2142G mutation. *MboII *digestion of the PCR products of representative samples was displayed on 2% agarose gel. The 289 bp A2142G-positive PCR products of *2142G *were cleaved into an 182 bp and a 107 bp fragments. The PCR product of GJ2040 was cleaved into a 164 bp and a 125 bp fragments; and the product of GJ2111 was cleaved into 245 bp and 44 bp fragment(s) (**C**). The A2142G and other mutations were confirmed by sequencing (**D**). Two new *MboII*-sensitive sequences were characterized as CTTCA (2222–2226) for GJ2040 and GAAG (2081–2084) for GJ2111.

To detect the prevalence of CLR^r ^*H. pylori *infection, a *BsaI*-restriction PCR-RFLP assay was developed using the PCR product of *23S rRNA *for detection of the A2143G mutation as illustrated in Fig 1. The A2143G mutation was detected in 65 of the 458 PCR products, which showed the presence of *H. pylori*, by the RFLP assay (Fig 2A). In 41 of the 65 A2143G-positive cases (63.1%), both the *BsaI*-sensitive and -insensitive PCR products of *23S rRNA *were observed. For 12 representative samples, results of the PCR-RFLP assay were consistent with that of sequencing (Fig 2B).

To detect the A2142G mutation within *23S rRNA*, a *MboII*-restriction PCR-RFLP assay was also developed (Fig 1). No A2142G point mutation was observed among 458 subjects with *H. pylori *infection. However, two new *MboII*-RFLP patterns were detected in DNA samples from 17 subjects (Fig 2C): a pattern consisting of a 164 bp and a 125 bp fragment was observed from 11 subjects and another pattern consisting of a 245 bp and 44 bp fragment was detected from 6 cases. Sequencing showed that the former 11 cases had a sequence change of AACC → GAAG (2081–2084) and the latter 6 cases had a sequence change of AAGGG → CTTCA (2222–2226) (Fig 2D).

To investigate the relationship between the prevalence of CLR^r ^*H. pylori *infection and administration of CLR as a component of an optimal triple therapy, A2143G mutation rates within *23S rRNA *in each therapy group were analyzed (Table 1). The presence of the A2143G mutation in the OAC group was 3.1 times higher than that in the OA group [31.7% vs. 10.2%, p = 0.0005; OR 4.09 (95% CI = 1.64–10.23)] and 2.3 times higher than that in the placebo group [31.7% vs. 13.8%, p = 0.0040; OR 2.89 (95% CI = 1.28–6.46)]. The difference of the A2143G mutation rate due to CLR was 21.5% between the OAC and OA groups. In the OAC group, the co-infection rate (14.6%) of *BsaI*-insensitive *H. pylori *among subjects infected with *BsaI*-sensitive *H. pylori *was higher than those in the OA group (6.4%) and the placebo group (9.6%) but not significant (p = 0.0838 and 0.3258, respectively). Moreover, the sequence change AAGGG → CTTCA (2222–2226) in the OAC group was 12.3 times of the placebo group (4.9% vs. 0.4%, p = 0.0497; OR 13.28, 95% CI 0.91–379); the AACC → GAAG (2081–2084) in the OAC group was 11.2 times of the OA group (14.6.7% vs. 1.3%, p = 0.018; OR 8.38, 95% CI 1.25–68.84) and 7.7 times of the placebo group (14.6% vs. 1.9%, p = 0.002; OR 5.51, 95% CI 1.18–25.12) (Table 1).

## Discussion

Among Chinese subjects enrolled in the long term population-based trial [10], we found that the proportion of the primary CLR^r ^related A2143G mutation among *23S rRNA*-positive subjects in the placebo group was 13.8% (36/260), which was similar to the average reported CLR-resistant prevalence of 10.5% (35/334) within China: 5.2% (5/96) [14], 9.8% (4/41) [13], 13.5% (12/89) [15], and 14.8% (16/108) [16]. That the CLR^r ^infection rate in the OA group was slightly lower than that in the placebo group (p = 0.274) indicated that the OA treatment for eradication of *H. pylori *might reduce the prevalence of primary CLR^r ^among the treated subjects. Heep *et al*. reported that secondary resistance to CLR was up to 50% in a study of 554 *H. pylori *isolates after failure of therapy [17]. It is well recognized that eradication therapy with CLR led to the prevalence of CLR^r ^*H. pylori *infection [8,9]. In the present study, we observed a 31.7% A2143G mutation rate among *H. pylori *infected subjects three years after treatment with CLR, which is significantly higher than that among subjects without CLR treatment in the OA and placebo groups (OR = 4.09 and 2.89, respectively). An optimal way to validate the exact effect of CLR use on the formation of the secondary CLR resistance was to compare the prevalence of the A2143G mutation among subjects before and after administration of CLR. Because of the lack of gastric juice samples before eradication therapy, the original mutation status of *23S rRNA *in samples was not available, thus such comparison could not be carried out in the present study. This is a limitation of our study. The strength of this study was that all of the subjects in the OAC group had the same CLR treatment history in 2000, and those in the OA and placebo group did not. Therefore, the prevalence of CLR^r ^*H. pylori *infection among subjects with and without CLR use in the local area was reliably obtained. These results were useful for evaluation of the exact effect of administration of CLR for eradication of *H. pylori *on the prevalence of the A2143G mutation among Chinese subjects.

Unlike indirect assays such as ELISA and ^13^C-UBT, PCR assays are direct assays to detect *H. pylori *infection. Thus, these assays should result in higher specificity than the indirect assays. *H. pylori *colonizes on the apical surface of stomach's epithelial cells and sheds into gastric juice with the regular renewal of gastric mucosa. Several PCR assays based on *H. pylori *DNA from gastric juice/biopsy sample were reported for detection of *H. pylori *infection [13,18,19]. The possible *H. pylori*-specific target genes included *ureA*, *ureB*, *ureC*, *ureD*, *16S rRNA*, and *23S rRNA*. Point mutations of *23S rRNA *were closely related to formation of CLR resistance [8,11,12]. Because the PCR product of *23S rRNA *described in this study could be used as a biomarker to detect *H. pylori *infection and CLR resistance simultaneously, this assay has advantages over other PCR-based methodologies. However, the specificity and sensitivity of the *23S rRNA *PCR assay was not evaluated extensively. No single test is accepted as the standard for diagnosis of *H. pylori *infection [20,21]. In the present study, the status of *H. pylori *infection was detected by the *23S rRNA *PCR assay, ^13^C-UBT, and ELISA for each subject, which provided an opportunity to assess the specificity and sensitivity of these assays. As expected, the highest specificity was observed for the *23S rRNA *PCR assay when both CUBT and ELISA assays were negative for *H. pylori *infection. The Youden's index of the *23S rRNA *PCR assay was similar to that of the ELISA assay but lower than that of ^13^C-UBT (Table 2).

Generally, PCR-based assays used to detect *H. pylori *infection are considered the most sensitive. However, the sensitivity of the *23S rRNA *PCR assay was lower than ^13^C-UBT and ELISA in the present study (Table 2). Because the genomic DNA could not be extracted from gastric juice samples immediately after collection and that the shedded gastric epithelial cells easily self-decompose, the quality and amount of DNA extracted is variable between samples. In this study, fixative was added to gastric juice samples at the local clinics to prevent degeneration of DNA, and then the samples were stored at -20°C. These samples were shipped to the laboratory using dry ice and stored at -80°C. Although the fixative was removed with repeated washings before DNA extraction, it is possible that the quality of the DNA was adversely affected. The fixation process could be omitted if gastric juice samples can be stored at -80°C immediately after collection. However, it would be optimal if the DNA were isolated from fresh samples immediately after collection.

CLR^r ^results mainly from point mutations in the peptidyltransferase loop region of *23S rRNA *[8,9,11-13]. The most frequent mutations are A2143G and A2142G [8]. Other mutations such as A2142C, A2115G, G2141A, and T2717C might also be associated with CLR^r^. Recent reports demonstrated that the A2143G point mutation of *23S rRNA *were detected in average of 90% (44 of 49) CLR^r ^*H. pylori *isolates from Chinese subjects [13-16,22]. In the present study, the A2143G mutation was detectable in 65 of 458 *23S rRNA *PCR products by the *BsaI*-restriction PCR-RFLP assay (Table 1). No A2142G mutation was detected in these samples. These results suggest that the A2143G may be a very useful CLR^r ^indicator for selection of optimal therapy against *H. pylori *infection in China.

In 41 of the 65 A2143G-positive cases (63.1%), both the *BsaI*-sensitive and -insensitive *23S rRNA *PCR products was observed. Because the PCR products of the A2143G-positive control (CLR^r^-1) were digested by *BsaI *enzyme completely in each experiment, partial restriction could be excluded. Thus, multi-strain *H. pylori *infection likely happened among these cases. Sequencing results of 12 representative samples supported the deduction (Fig. 2B). Isolation of the sensitive and resistant strains from cultures might provide a direct evidence to show the mixed colonization. Because the experimental conditions at the local clinics in Linqu County was not adequate to support primary *H. pylori *culture, isolation of *H. pylori *from biopsies of stomach was not carried out in the clinical trial. The high *H. pylori *infection rate in the local area might contribute to the high co-infection rate of the CLR sensitive and resistant *H. pylori *strains.

In addition, two new *MboII*-RFLP patterns were discovered in 17 subjects in the present study. Sequencing showed that 6 of the 17 cases had a sequence change of AAGGG → CTTCA (2222–2226) and that the remaining 11 cases had a sequence change of AACC → GAAG (2081–2084). The A2143G mutation was detected only in 3 of the 15 GAAG-positive cases (20%). Both the AAGGG → CTTCA (2222–2226) and AACC → GAAG (2081–2084) alterations are located around the conserved domain V of *23S rRNA *(2126–2712, equal to *Escherichia coli *numbering 2042 to 2628), a region closely related to resistance formation of macrolide antibiotics including CLR [23,24]. It was reported that the point mutation A2224G might not contribute to the CLR resistance among Japanese [27]. In the present study, we observed that the presence of the AAGGG → CTTCA (2222–2226) and AACC → GAAG (2081–2084) sequence alterations in OAC group were significantly higher than those in the OA and placebo groups (Table 1). The A2143G mutation was detected only in 2 of the 11 AACC → GAAG (2081–2084) cases (18.2%). Whether these alterations contribute to CLR resistance should be investigated further.

## Conclusion

The primary prevalence of the CLR^r^-related A2143G mutation within the *23S rRNA *gene as determined by a novel PCR-RFLP assay is approximately 10~14% among Chinese subjects without CLR use history. Application of CLR-containing regimen for *H. pylori *eradication could increase risk of prevalence of the A2143G mutation significantly (OR = 3~4).

## Methods

### Study population and collection of gastric juice samples

All subjects were from those enrolled in the intervention trial. The experimental design and population of this intervention trial (trial no. NCI-OH-95-C-N029) have been described in detail elsewhere [10]. Fasting gastric juice samples were taken by a side-tube connected to vacuum device within the gastroendoscope at the end of the intervention trial in 2003. All subjects with more than 5 mL of gastric juice were eligible for the present study. Seven hundred and seventy of gastric juice samples were collected during the endoscope examination. In the OA group, 307 subjects with *H. pylori *seropositive were given amoxicillin (1000 mg) and omeprazole (20 mg) to take twice daily for two weeks in 1995, while 310 subjects with seropositive were given placebo capsules as described [10]. One hundred and fifty three subjects with *H. pylori *seronegative at baseline but were subsequently determined to be ^13^C-UBT-positive received a first-line triple therapy of CLR (500 mg), amoxicillin (1000 mg) and omeprazole (20 mg) to take twice daily for two weeks, according to instruction of the Ethic Committee in 2000. Status of *H. pylori *infection of each individual was detected by ^13^C-UBT and ELISA assays [25,26].

### Extraction of genomic DNA from gastric juice samples

To avoid self-decomposition, the shedded gastric mucosa was fixed immediately after collection of the samples at a local clinic in Linqu County. All the collected gastric juice specimens (5–30 mL) were centrifuged at 10,000 *g *for 10 min. After centrifugation, the supernatant liquid was removed leaving ~1 mL residue. The sample was then resuspended in 2 mL of FAA fixative (ethanol, acetic acid, and 40% formaldehyde at volume proportion of 9:1:1) and frozen at -20°C. The collected samples were shipped with dry ice and stored at -80°C at Etiology Laboratory, Beijing Institute for Cancer Research, Beijing.

For DNA extraction, the frozen samples were thawed, and the fixative was removed with 2 × 5 mL aliquots of PBS. The samples were centrifuged at 10,000 *g *for 10 min and the supernatant liquid was removed after each PBS wash. The sediment was then resuspended in 5 mL of 10% SDS containing hyaluronidase (final concentration, 20 U/mL) (Sigma, Inc, USA) and incubated at 37°C for two hours to digest mucus. Proteinase K (final concentration, 20 U/mL) (Merck, Inc, Germany) was then added, and the sample was incubated at 55°C for more than three days until the desquamated tissue was digested completely. The DNA in the samples was extracted following a standard phenol chloroform procedure.

### Amplification of the *H. pylori*-specific 23S rRNA by PCR

A 289 bp fragment of *H. pylori *chromosomal DNA was amplified by PCR as reported recently [13]. Briefly, the *23S rRNA *gene of *H. pylori *[GenBank: U27270] was amplified using the following primers: 5'-GCA TGA ATG GCG TAA CGA GAT G-3' and 5'-CCC AGT CAA ACT ACC CAC CAA G-3' (corresponding to 2049–2070 and 2316–2337 of the *23S rRNA *gene, respectively). The 289 bp PCR products were verified on 2% agarose gel.

### RFLP assays

The 289 bp amplicon of *23S rRNA *was digested with the restriction enzymes *BsaI *and *MboII *(New England Biolabs, USA) in order to detect A2143G and A2142G point mutations, respectively (Fig 1) [13,27]. An 8% PAGE gel was used to observe the restriction products. The amplicon of *H. pylori 26695 *strain was used as negative control. The CLR^r^-1 strain selected from *H. pylori 26695 *[by CLR] contained an A2143G point mutation and was used as one positive control [13]. An A2142G point mutation was introduced into the amplicon of *H. pylori 26695 *by mutation PCR. One of the A2142G clones (confirmed by sequencing) was used as another positive control.

### DNA sequencing

PCR products of *H. pylori 23S rRNA *were sequenced by an ABI Prism 377 sequencer (PE Biosystems, USA) with fluorescent dye terminators.

### Statistical analysis

For statistical analysis, the χ^2 ^test was used to evaluate the significance of differences of *H. pylori *infection and CLR^r ^frequency between CLR and control groups. All p-values were two-sided and p < 0.05 was considered statistically significant. All analyses were performed with SAS software version 8.0.

## Authors' contributions

ZL carried out experiments including DNA extraction, detection of 23S rRNA and point mutations by PCR-RFLP. JS setup the PCR-RFLP assay. LZ carried out the ELISA and ^13^C-UBT assays. LS collected the gastric juice samples by endoscope. QL validated the quality of DNA from the samples. BZ, JZ, and LG pretreated the samples in the local clinic. GF performed the statistical analysis. JM collected patients' followup data. W-CY conceived the idea of the intervention trial and advised on the manuscript. DD conceived the idea of the present study, analyzed the data, and wrote the manuscript.
